# Phenylthiophenecarboxamide Antagonists of the Olfactory Receptor Co-Receptor Subunit from a Mosquito

**DOI:** 10.1371/journal.pone.0084575

**Published:** 2013-12-17

**Authors:** Sisi Chen, Charles W. Luetje

**Affiliations:** Department of Molecular and Cellular Pharmacology, University of Miami Miller School of Medicine, Miami, Florida, United States of America; Plant and Food Research, New Zealand

## Abstract

Insects detect environmental chemicals using chemosensory receptors, such as the ORs, a family of odorant-gated ion channels. Insect ORs are multimeric complexes of unknown stoichiometry, formed by a common subunit (the odorant receptor co-receptor subunit, Orco) and one of many variable subunits that confer odorant specificity. The recent discovery of Orco directed ligands, including both agonists and antagonists, suggests Orco as a promising target for chemical control of insects. In addition to competitively inhibiting OR activation by Orco agonists, several Orco antagonists have been shown to act through a non-competitive mechanism to inhibit OR activation by odorants. We previously identified a series of Orco antagonists, including N-(4-ethylphenyl)-2-thiophenecarboxamide (OX1a, previously referred to as OLC20). Here, we explore the chemical space around the OX1a structure to identify more potent Orco antagonists. Cqui\Orco+Cqui\Or21, an OR from *Culex quinquefasciatus* (the Southern House Mosquito) that responds to 3-methylindole (skatole) and is thought to mediate oviposition behavior, was expressed in *Xenopus* oocytes and receptor function assayed by two-electrode voltage clamp electrophysiology. 22 structural analogs of OX1a were screened for antagonism of OR activation by an Orco agonist. By varying the moieties decorating the phenyl and thiophene rings, and altering the distance between the rings, we were able to identify antagonists with improved potency. Detailed examination of three of these compounds (N-mesityl-2-thiophenecarboxamide, N-(4-methylbenzyl)-2-thiophenecarboxamide and N-(2-ethylphenyl)-3-(2-thienyl)-2-propenamide) demonstrated competitive inhibition of receptor activation by an Orco agonist and non-competitive inhibition of receptor activation by an odorant. The ability to inhibit OR activation by odorants may be a general property of this class of Orco antagonist, suggesting that odorant mediated behaviors can be manipulated through Orco antagonism. The high conservation of Orco across insect species and previous demonstrations that various Orco ligands are active at ORs derived from several different insect orders suggests that Orco antagonists may have broad applicability.

## Introduction

The interactions of insects with humans can be beneficial (pollination of crops), as well as detrimental (disease transmission, crop destruction). Many insect behaviors, such as feeding, mating and oviposition, are driven by olfaction, making insect olfactory receptors appealing targets for insect control strategies [1]. The OR class of insect olfactory receptors are a novel class of ligand (odorant) gated cation channel [2,3], located on the dendrites of olfactory sensory neurons in the antennae. ORs are composed of a common subunit (the odorant receptor co-receptor subunit, known as Orco [4]) that is highly conserved across species and a variable subunit that confers odorant specificity [5-12]. The specificity subunits are thought to mediate odorant recognition because changing this subunit alters odorant preference [13-15] and mutations in a specificity subunit can alter odorant sensitivity [16,17]. Both Orco and the specificity subunit are thought to contribute to the structure of the ion channel pore [2,18,19]. However, the number and stoichiometry of these subunits in a functional OR is currently unknown. These receptors also may initiate, or be modified by, second messenger cascades [3,20,21].

Insect ORs are not related to the receptors and channels of humans and other tetrapods [5], suggesting that control of detrimental insect activity can be achieved, while minimizing environmental toxicity, through the development of insect OR selective compounds. One approach to developing these compounds involves the identification of particular specificity subunits that mediate recognition of behaviorally important odorants [13,15,22-24], followed by extensive ligand screening [25,26]. However, high diversity among the specificity subunit repertoires of different species, as well as variation in the odorants and specificity subunits that are important for species-specific behaviors, makes this approach exceptionally labor intensive [1,27]. The development of compounds active at multiple ORs across different species would be more useful. 

The recent identification of VUAA1, an agonist of the Orco subunit [25], suggests Orco-directed compounds as a promising new direction for the development of insect repellants and additional agonists were subsequently identified [28-30]. Orco agonists identified to date are closely related to VUAA1, suggesting a restrictive set of structural requirements for Orco agonism. A larger, more diverse series of compounds can competitively antagonize Orco agonist activity [29,31]. Importantly, several of these Orco antagonists were shown to inhibit odorant activation of ORs through a non-competitive mechanism. 

## Results and Discussion

**Figure 1:**
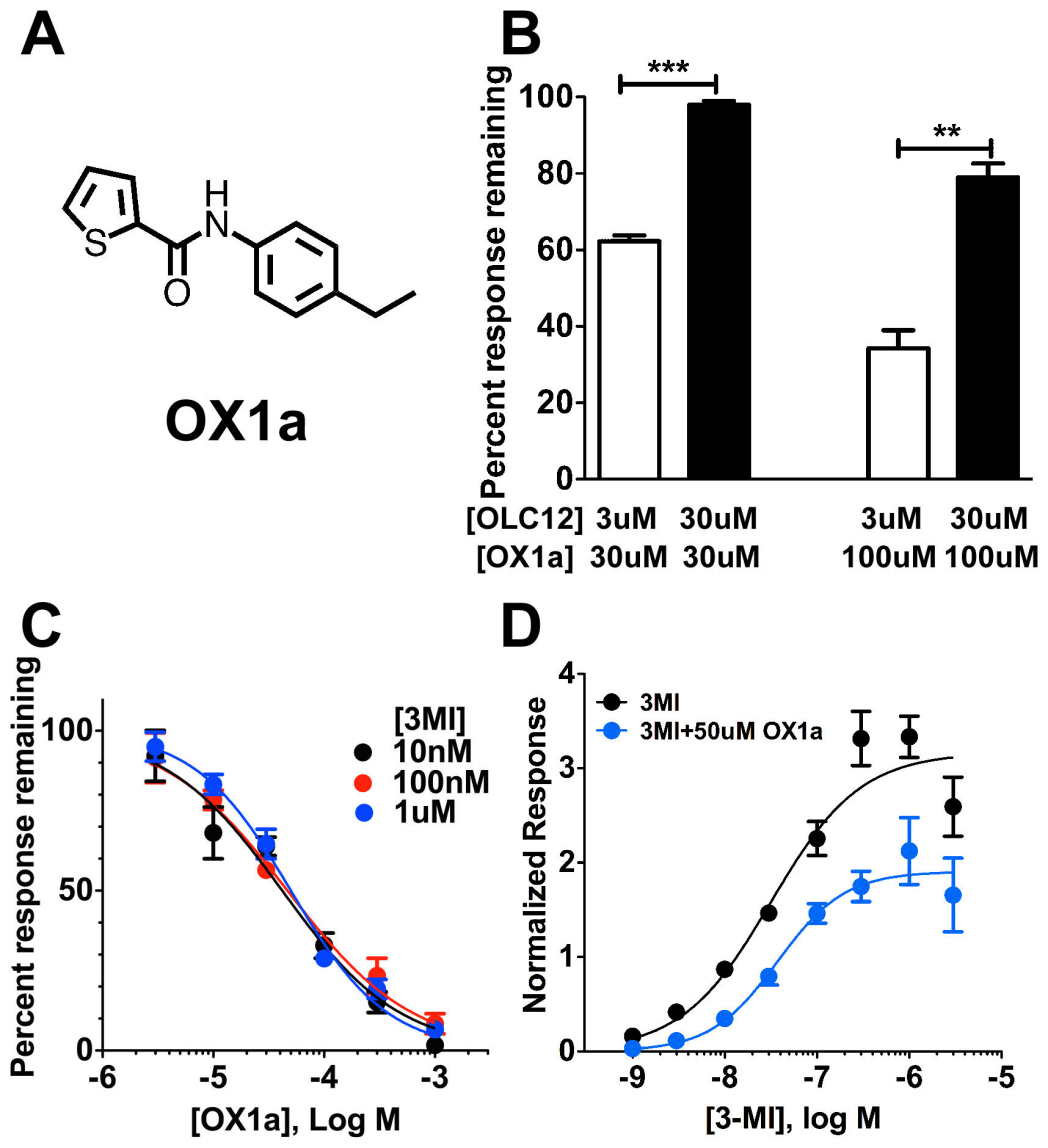
Non-competitive inhibition of odorant activation of an insect OR by an Orco antagonist. **A**. Structure of N-(4-ethylphenyl)-2-thiophenecarboxamide (OX1a). **B**. Increasing the concentration of Orco agonist (OLC12) decreases the effectiveness of OX1a. Oocytes expressing Cqui\Orco+Cqui\Or21 were activated with 3 µM or 30 µM OLC12, in the absence or presence of 30 µM or 100 µM OX1a. Responses in the presence of OX1a are presented as a percentage of the average of the two preceding responses to OLC12 alone (mean ± SEM, n=3). Statistical significance: **p<0.01, ***p<0.001 (two-tailed, unpaired t-test). **C**. OX1a inhibition of Cqui\Orco+Cqui\Or21 activation by 3-methylindole (3MI) is not altered by changes in odorant (3MI) concentration. IC_50_ values for OX1a inhibition of responses to 10nM 3MI (47 ± 5 µM), 100nM 3MI (42 ± 7 µM) and 1uM 3MI (50 ± 4 µM) did not differ (p=0.2970, F-test). **D**. Co-application of 50 µM OX1a significantly reduces the maximal response to 3MI, as compared to the response to 3MI in the absence of OX1a (p<0.0001, F-test). The EC_50_ for 3MI activation of Cqui\Orco+Cqui\Or21 in presence of 50 µM OX1a (37 ± 7 nM) did not differ (p=0.57, F-test) from the EC_50_ for activation of Cqui\Orco+Cqui\Or21 by 3MI alone (32 ± 7 nM).

While many of the previously identified Orco antagonists are large structures that are unlikely to be useful as repellants, our previous screen [29] identified several Orco antagonists of smaller size, such as N-(4-ethylphenyl)-2-thiophenecarboxamide (Figure 1A), suggesting a promising starting point for the identification of new Orco ligands. We previously referred to this compound as OLC20 (Orco Ligand Candidate 20). Here, we use "OX" to denote Orco antagonists and will refer to this compound OX1a. We previously demonstrated that a larger Orco antagonist (OLC15) could non-competitively antagonize odorant activation of insect ORs [29]. However, whether OX1a also possesses this useful functional property was not tested. 

In Figure 1, we examined the antagonist properties of OX1a against Cqui\Orco+Cqui\Or10, an OR from the Southern House Mosquito (*Culex quinquefasciatus*) that is activated by 3-methylindole, an oviposition attractant [22]. Cqui\Or10 has been renamed Cqui\Or21, as a result of recent annotation of the *Cx. quinquefasciatus* genome [32], and we are adopting the new nomenclature here. This OR was expressed in *Xenopus* oocytes (see Methods) and activated with OLC12 (2-((4-ethyl-5-(4-pyridinyl)-4H-1,2,4-triazol-3-yl)sulfanyl)-N-(4-isopropylphenyl)-acetamide), an Orco agonist we previously identified [29]. We measured blockade of Cqui\Orco+Cqui\Or21 achieved by OX1a when the Orco agonist (OLC12) concentration was increased from 3µM to 30µM (Figure 1B). OX1a, at two concentrations (30 and 100µM), was significantly less effective at inhibiting the response to the higher concentration of OLC12. These results, together with our previous findings [29], indicate that OX1a is a competitive antagonist of Orco. To determine whether OX1a could also inhibit odorant activation of Cqui\Orco+Cqui\Or21 through a non-competitive mechanism, we generated concentration-inhibition curves for OX1a inhibition of activation by three concentrations of 3-methylindole. IC_50_ values for OX1a inhibition of receptor activation by 10nM, 100nM, and 1µM 3-methylindole were not significantly different (Figure 1C), suggesting a non-competitive mechanism for OX1a inhibition of odorant activation. Comparison of the concentration-response relationship for 3-methylindole activation of Cqui\Orco+Cqui\Or21 in the absence and presence of 50 µM OX1a showed that while the EC_50_ for activation was unchanged, the maximal response was significantly reduced by the presence of OX1a (Figure 1D). These results with OX1a, together with our previous findings with OLC15 [29] and the work of Jones et al. [31], suggest that inhibition of odorant activation through a non-competitive mechanism may be a general property of VUAA1-like and phenylthiophenecarboxamide Orco antagonists.

**Figure 2:**
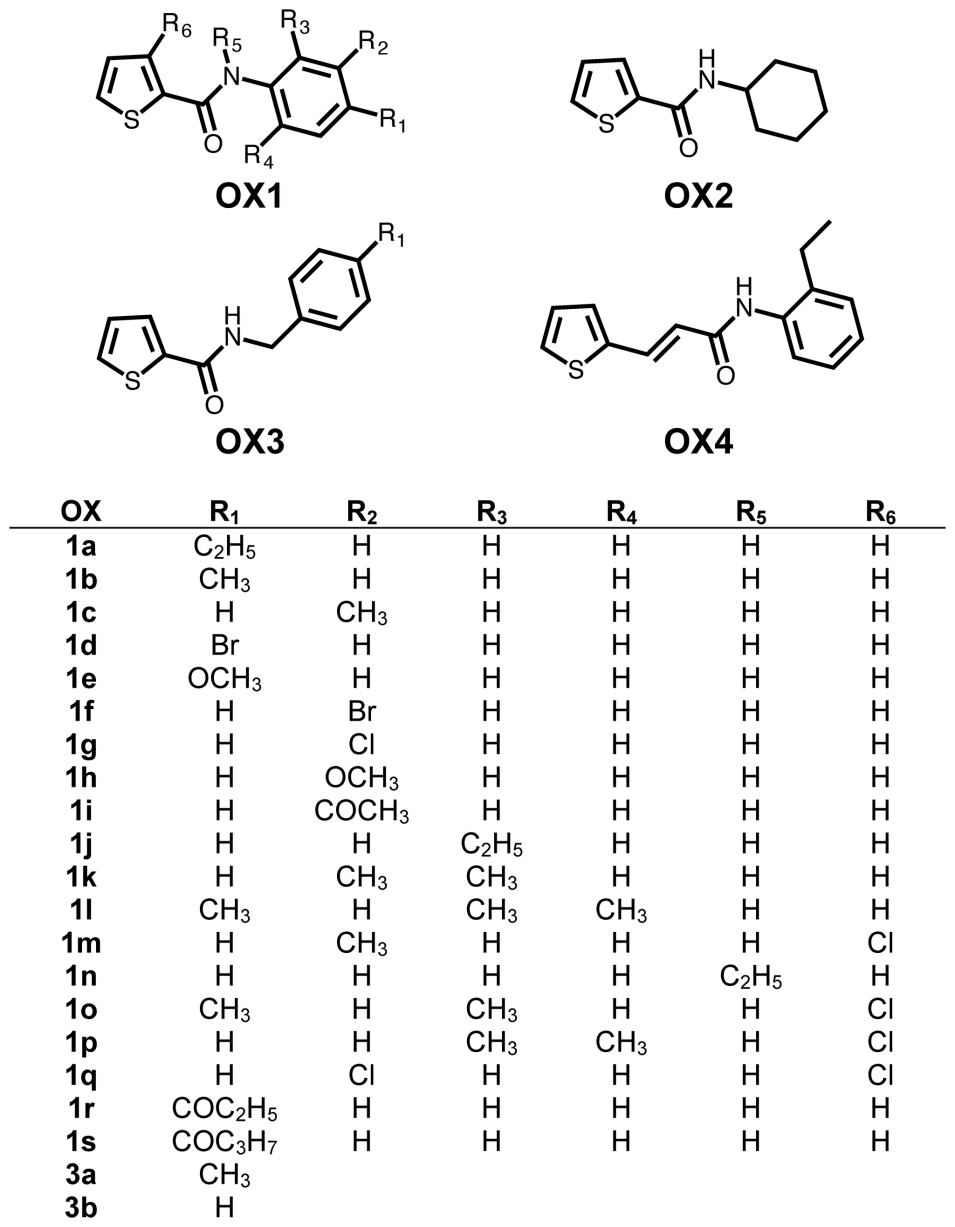
Compounds tested in this study.

**Figure 3:**
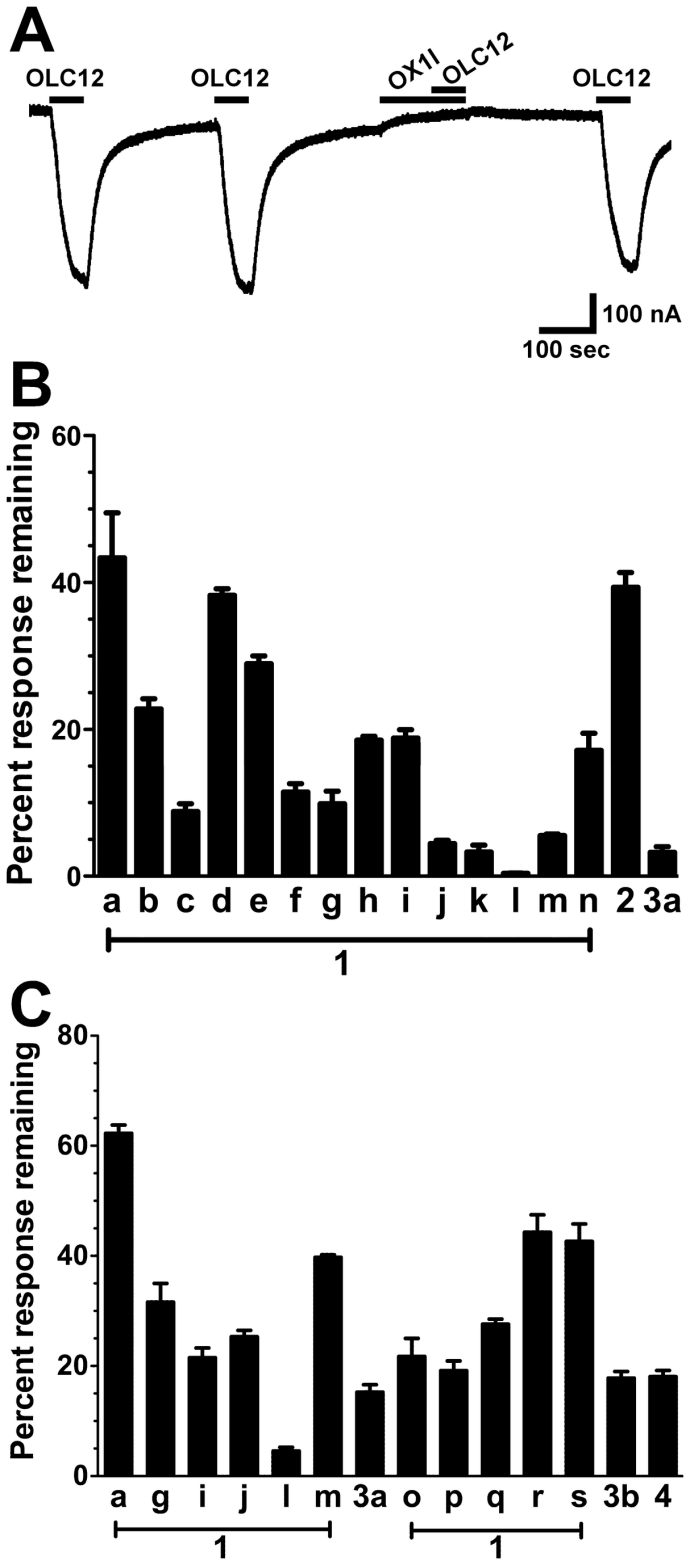
A screen of OX1a analogs for Orco antagonist activity. **A**. An example of the antagonist screening protocol. An oocyte expressing Cqui\Orco+Cqui\Or21 was exposed to 60 sec applications of 3 µM OLC12 with 4 min washed between applications. 100 µM OX1l was applied for 90 sec preceding the third application of OLC12 and then co-applied during the OLC12 application. **B**. Responses of Cqui\Orco+Cqui\Or21 to 3 µM OLC12 (~EC_25_) in the presence of 100 µM of each analog are presented as a percentage of the average of the two preceding responses to OLC12 alone (mean ± SEM, n = 3-7). Inhibition by OX1d and OX2 did not differ from inhibition by OX1a. Inhibition by all other compounds was significantly different from inhibition by OX1a: OX1e (p<0.05); OX1b-c,f-n, OX3a (p<0.001). **C**. A screen of additional OX1a analogs for Orco antagonist activity. Responses of Cqui\Orco+Cqui\Or21 to 3 µM OLC12 in the presence of 30 µM of each candidate antagonist are presented as a percentage of the average of two preceding responses to OLC12 alone (mean ± SEM, n=3-5). Inhibition by all compounds was significantly different from inhibition by OX1a (p<0.001). Inhibition by OX1i was significantly different (p<0.001) from values for OX1r and OX1s. Inhibition by OX1l was significantly different from values for OX1o (p<0.001) and OX1p (p<0.05). Inhibition by OX1m was significantly different (p<0.001) from values for OX1o and OX1p. Inhibition values that did not differ are: OX1g and OX1q, OX1j and OX4, OX1m and OX1q.

To identify additional Orco antagonists, we screened a panel of 15 compounds (OX1b-OX1n, OX2, OX3a) structurally related to OX1a (Figure 2). Cqui\Orco+Cqui\Or21 was activated with 3 µM OLC12, the EC_25_ (the EC_50_ for OLC12 activation of Cqui\Orco+Cqui\Or21 was 6.6 ± 1.4 µM, n_H_ = 1.42 ± 0.32). 100 µM of each compound was tested for the ability to inhibit the OLC12 response (Figure 3B). Each of the 15 compounds was able to inhibit OLC12 activation of Cqui\Orco+Cqui\Or21, with many compounds displaying significantly greater inhibition than OX1a. Only two alterations failed to significantly improve antagonist activity: changing the 4-ethylphenyl portion of OX1a to an undecorated cyclohexyl ring (OX2) and a 4-bromo substitution on the phenyl ring (OX1d). Interestingly, while the 4-bromo substitution failed to improve antagonist activity, the 3-bromo (OX1f) and 3-chloro (OX1g) substitutions did show significant improvement. Changing the size, position or number of alkyl moieties on the phenyl ring with a 2-ethyl (OX1j), 4-methyl (OX1b), 3-methyl (OX1c), 2, 3-dimethyl (OX1k) or 2, 4, 6-trimethyl (OX1l) phenyl ring, all resulted in significantly improved antagonist activity. Changing the 4-ethyl group (OX1a) to 4-methoxy (OX1e), 3-methoxy (OX1h) or 3-acetyl (OX1i) also significantly improved antagonist activity. Addition of a chloro- group to the thiophene of OX1c (OX1m), insertion of a carbon between the nitrogen and the phenyl ring of OX1b (OX3a) and movement of the ethyl group from the phenyl ring to the nitrogen (OX1n) also yielded improved antagonist activity. 

To further explore the structural features of Orco antagonists, we screened an additional 7 compounds (OX1o-OX1s, OX3b, OX4) (Figure 3C) chosen based on the structures of OX1g, OX1i, OX1j, OX1l, OX1m and OX3a. We used the same inhibition protocol as in panel B, but tested the compounds at 30µM to allow better resolution of potent antagonists. Insertion of an ethene between the thiophene and the carboxamide of OX1j (OX4), removal of the 4-methyl from the phenyl ring of OX3a (OX3b), or addition of a chloro to the thiophene ring of OX1g (OX1q) were well tolerated. Addition of a chloro to the thiophene ring and removal of a methyl from the phenyl ring of OX1l (OX1o, OX1p) resulted in a significant loss of antagonist activity. Changing the 3-acetyl on the phenyl ring of OX1i to a 4-propionyl (OX1r) or a 4-butyryl (OX1s) also resulted in a significant loss of antagonist activity.

**Figure 4:**
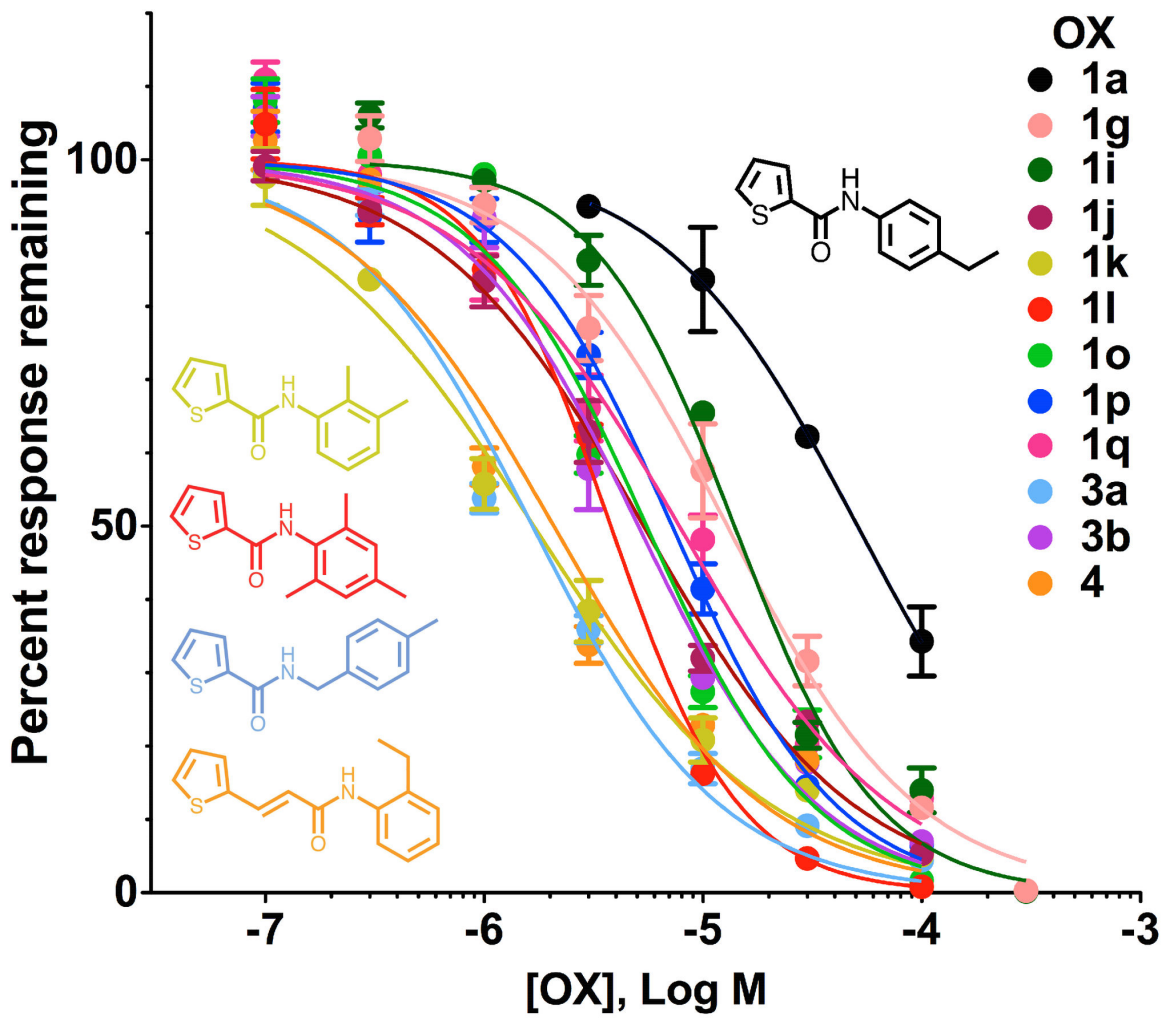
Concentration-inhibition curves for OX1a, OX1g, OX1i, OX1j, OX1k, OX1l, OX1o, OX1p, OX1q, OX3a, OX3b and OX4 inhibition of Cqui\Orco+Cqui\Or21 activated by 3 µM OLC12. IC_50_ and n_H_ values may be found in Table 1.

The data presented in Figure 3 suggested that many of the tested compounds were more potent antagonists than OX1a. To quantitatively evaluate this improvement in antagonist potency, we constructed concentration-inhibition curves for eleven of these compounds (Figure 4). These compounds displayed increases in potency of 3.6-fold to 30-fold, with IC_50_ values for antagonism of OLC12 activation of Cqui\Orco+Cqui\Or21 ranging from 1.7 µM to 14 µM (Table 1), all a significant improvement over OX1a (IC_50_ = 51 ± 6 µM). Two of the four most potent compounds were from the OX1 series: OX1k, with methyl decorations at the 2 and 3 positions of the phenyl ring; OX1l, with methyl decorations at the 2, 4 and 6 positions of the phenyl ring. The other two compounds were OX3a, with a carbon insertion between the carboxamide and the phenyl ring, and OX4, with an ethene insertion between the thiophene ring and the carboxamide. The structures of these compounds are shown in Figure 4. 

**Table 1:** IC_50_ and n_H_ values for Orco antagonists.

**Compound**	**IC_50_ (µM)**	**n_H_**
OX1a	51 ± 6	1.0 ± 0.1
OX1g	13 ± 1	1.0 ± 0.1
OX1i	14 ± 1	1.3 ± 0.1
OX1j	5.4 ± 0.4	0.9 ± 0.1
OX1k	1.7 ± 0.2	0.8 ± 0.1
OX1l	3.8 ± 0.3	1.5 ± 0.1
OX1o	5.5 ± 0.7	1.1 ± 0.1
OX1p	7.2 ± 0.6	1.2 ± 0.1
OX1q	7.8 ± 0.9	0.9 ± 0.1
OX3a	1.7 ± 0.2	1.0 ± 0.1
OX3b	5.1 ± 0.5	1.1 ± 0.1
OX4	2.1 ± 0.3	0.9 ± 0.1

Cqui\Orco+Cqui\Or21 was activated by 3 µM OLC12. The IC_50_ values for inhibition by each compound were significantly different from the IC_50_ for inhibition of OX1a (p<0.001, F-test).

**Figure 5:**
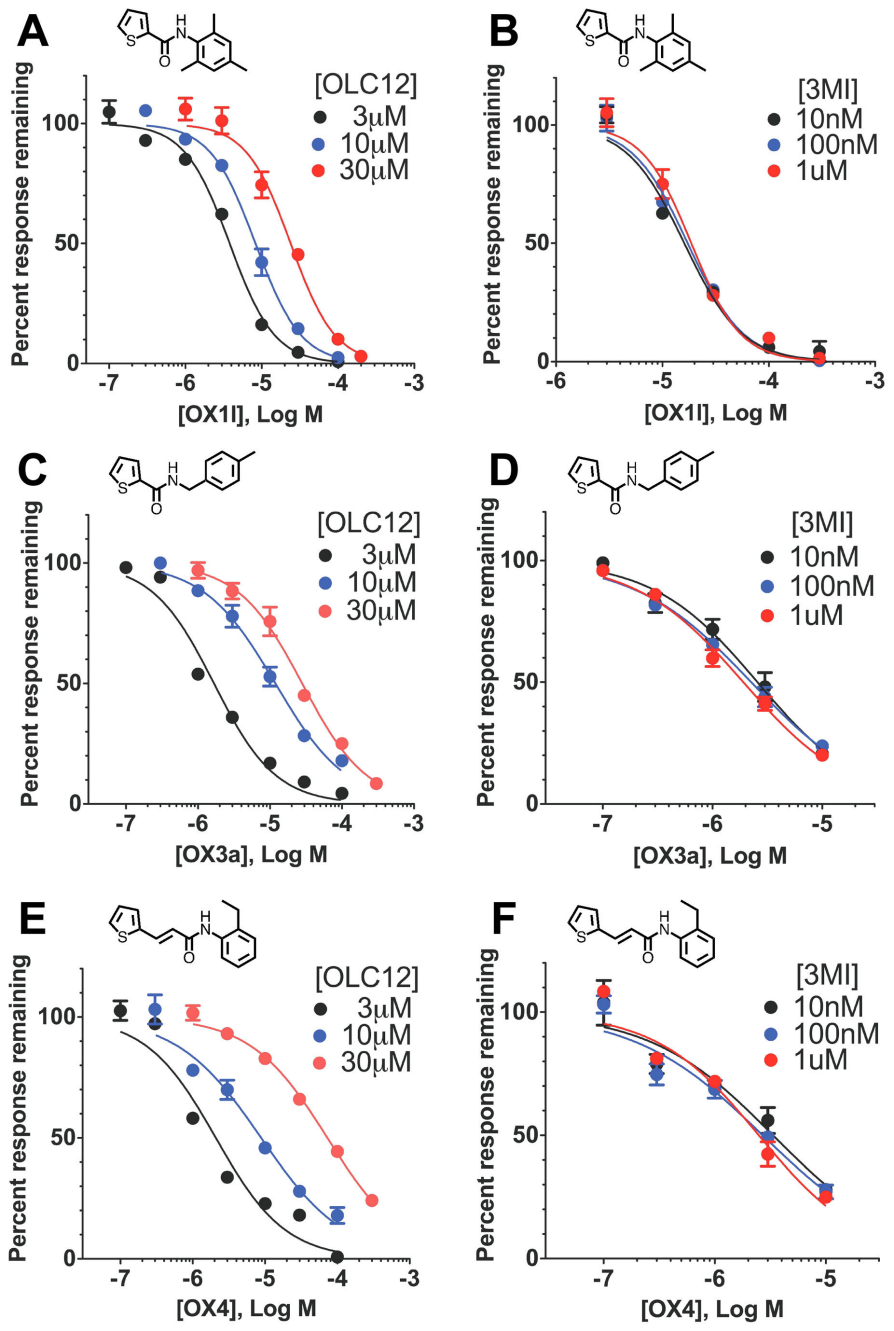
The potent Orco antagonists OX1l, OX3a and OX4 non-competitively inhibit odorant activation of a mosquito OR. **A**. Altering the concentration of Orco agonist (OLC12) shifts the OX1l inhibition curve. The IC_50_ for OX1l inhibition of Cqui\Orco+Cqui\Or21 activation by 10 µM OLC12 (8.3 ± 0.5 µM, n = 4) is significantly different from the IC_50_ for OX1l inhibition of Cqui\Orco+Cqui\Or21 activation by 3 µM OLC12 (3.8 ± 0.3 µM, n = 9) (p<0.0001, F-test). The IC_50_ for OX1l inhibition of Cqui\Orco+Cqui\Or21 activation by 30 µM OLC12 (24 ± 3 µM, n = 6) is significantly different from the IC_50_ for OX1l inhibition of Cqui\Orco+Cqui\Or21 activation by 3 µM OLC12 (p<0.0001, F-test) and from the IC_50_ for OX1l inhibition of Cqui\Orco+Cqui\Or21 activation by 10 µM OLC12 (p<0.0001, F-test). **B**. Altering odorant (3MI) concentration fails to alter the inhibition curve for OX1l antagonism of Cqui\Orco+Cqui\Or21 activated by 3MI. The IC_50_ values for OX1l inhibition of responses to 10 nM 3MI (17 ± 1 µM, n = 3), 100 nM 3MI (16 ± 1 µM, n = 3), and 1µM 3MI (19 ± 2 µM, n = 3) did not differ (p=0.3605, F-test). **C**. Altering the concentration of Orco agonist (OLC12) shifts the OX3a inhibition curve. The IC_50_ for OX3a inhibition of Cqui\Orco+Cqui\Or21 activation by 10 µM OLC12 (12 ± 1 µM, n = 3) is significantly different from the IC_50_ for OX3a inhibition of Cqui\Orco+Cqui\Or21 activation by 3 µM OLC12 (1.7 ± 0.2 µM, n = 4) (p<0.0001, F-test). The IC_50_ for OX3a inhibition of Cqui\Orco+Cqui\Or21 activation by 30 µM OLC12 (28 ± 2 µM, n = 3) is significantly different from the IC_50_ for OX3a inhibition of Cqui\Orco+Cqui\Or21 activation by 3 µM OLC12 (p<0.0001, F-test) and from the IC_50_ for OX3a inhibition of Cqui\Orco+Cqui\Or21 activation by 10 µM OLC12 (p<0.0001, F-test). **D**. Altering odorant (3MI) concentration fails to alter the inhibition curve for OX3a antagonism of Cqui\Orco+Cqui\Or21 activated by 3MI. The IC_50_ values for OX3a inhibition of responses to 10 nM 3MI (2.6 ± 0.3 µM, n = 3), 100 nM 3MI (2.3 ± 0.2 µM, n = 4), and 1µM 3MI (1.9 ± 0.1 µM, n = 3) did not differ (p=0.07, F-test). **E**. Altering the concentration of Orco agonist (OLC12) shifts the OX4 inhibition curve. The IC_50_ for OX4 inhibition of Cqui\Orco+Cqui\Or21 activation by 10 µM OLC12 (8.8 ± 1.2 µM, n = 3) is significantly different from the IC_50_ for OX4 inhibition of Cqui\Orco+Cqui\Or21 activation by 3 µM OLC12 (2.1 ± 0.3 µM, n = 5) (p<0.0001, F-test). The IC_50_ for OX4 inhibition of Cqui\Orco+Cqui\Or21 activation by 30 µM OLC12 (73 ± 5 µM, n = 3) is significantly different from the IC_50_ for OX4 inhibition of Cqui\Orco+Cqui\Or21 activation by 3 µM OLC12 (p<0.0001, F-test) and from the IC_50_ for OX4 inhibition of Cqui\Orco+Cqui\Or21 activation by 10 µM OLC12 (p<0.0001, F-test). **F**. Altering odorant (3MI) concentration fails to alter the inhibition curve for OX4 antagonism of Cqui\Orco+Cqui\Or21 activated by 3MI. The IC_50_ values for OX4 inhibition of responses to 10 nM 3MI (3.4 ± 0.6 µM, n = 3), 100 nM 3MI (2.7 ± 0.4 µM, n = 3), and 1µM 3MI (2.5 ± 0.3 µM, n = 3) did not differ (p=0.42, F-test).

To determine whether these more potent Orco antagonists could exert an allosteric effect on activation of the receptor by odorant, we examined the properties of a representative of each structural class: OX1l, OX3a and OX4. First, we generated concentration-inhibition curves for each compound when inhibiting Orco activation by three different concentrations of OLC12, the Orco agonist (Figure 5A,C,E). In each case, the concentration-inhibition curves shifted rightwards when the concentration of OLC12 was increased from 3 µM to 30 µM and the IC_50_ values for the family of curves for each antagonist were significantly different. This result indicates a competitive mechanism for OX1l, OX3a and OX4 inhibition of OLC12. Next, we constructed concentration-inhibition curves for OX1l, OX3a and OX4 inhibition of odorant activation at three different concentrations of 3-methylindole, the odorant agonist (Figure 5B,D,F). For each antagonist (OX1l, OX3a or OX4), the concentration-inhibition curves were super-imposable, with the IC_50_ values for inhibition of activation by 10nM, 100nM or 1 µM 3-methylindole displaying no significant differences. These results demonstrate a non-competitive mechanism for OX1l, OX3a and OX4 inhibition of odorant activation of Cqui\Orco+Cqui\Or21, providing further support for the idea that inhibition of odorant activation through a non-competitive mechanism is a general property of VUAA1-like and phenylthiophenecarboxamide Orco antagonists.

The structural requirements for Orco agonism appear to be quite strict. While a few minor modifications to the original VUAA1 structure have resulted in the identification of additional Orco agonists, such as OLC12, most structural modifications resulted in a loss of agonist activity [28-30]. In some cases, these modifications generated Orco antagonists [29,31]. In contrast to what has been observed for agonists, our previous work [29] and the data we present here suggest that the chemical space for Orco antagonists is more densely populated with viable structures and is likely to be a fruitful area for compound development. While our current exploration of the phenylthiophenecarboxamide structure yielded Orco antagonists with improved potencies, these compounds do not show improvements in boiling point (BP, in °C at 760 mmHg) or vapor pressure (VP, in mmHg at 25°C) over the OX1a parent compound (BP = 285.0, VP = 0.003). OX1k (BP = 284.6, VP = 0.003) and OX1l (BP = 293.7, VP = 0.002) were similar to OX1a, while OX3a (BP = 451.6, VP = 0) and OX4 (BP = 459.4, VP = 0) were much worse than OX1a. The development of Orco antagonists with sufficient volatility to be useful as airborne repellants or "confusants" will clearly require additional effort.

The various Orco ligands identified to date have been shown to be active at ORs of insect species from several different orders, including Diptera (*D. melanogaster*, *A. aegypti, A. gambiae*, *C. quinquefasciatus*), Lepidoptera (*O. nubilalis*, *H. virescens*) and Hymenoptera (*H. saltator*) [25,28-31]. This broad activity is most likely due to the high degree of conservation among the Orco subunits of various insect species [6]. The ability of Orco antagonists to inhibit odorant activation of insect ORs through an allosteric mechanism (this study and [29,31] suggests that Orco antagonists can be developed into broad-spectrum compounds with which to manipulate insect behavior. This idea is supported by a recent study demonstrating that male and female *Aedes aegypti* mosquitoes lacking Orco have impaired food-seeking behavior, and that females have a reduced preference for humans over other mammals, as a blood meal source [33]. Of course, some selectivity may be desirable. Compounds that preferentially affect deleterious species, such as disease vectors, while not affecting more useful species, such as pollinators, would be highly desirable. Whether such selective compounds can be developed must await further study.

## Materials and Methods

### Materials

*Xenopus laevis* frogs were purchased from Nasco. The care and use of *Xenopus laevis* frogs in this study were approved by the University of Miami Animal Research Committee and meet the guidelines of the National Institutes of Health. Odorants, Orco ligands and other chemicals were from Sigma-Aldrich. Cqui\Or21 and Cqui\Orco were obtained as previously described [22,23] and inserted into the pGEMHE vector [34]. Cqui\Or10 was recently renamed Cqui\Or21, as a result of recent annotation of the *Cx. quinquefasciatus* genome [32].

Orco antagonists tested in this study (with CAS#, if available) were: OX1a, N-(4-ethylphenyl)-2-thiophenecarboxamide; OX1b, N-(4-methylphenyl)-2-thiophenecarboxamide; OX1c, N-(3-methylphenyl)-2-thiophenecarboxamide; OX1d, N-(4-bromophenyl)-2-thiophenecarboxamide; OX1e (64419-14-3), N-(4-methoxyphenyl)-2-thiophenecarboxamide; OX1f, N-(3-bromophenyl)-2-thiophenecarboxamide; OX1g, N-(3-cholorphenyl)-2-thiophenecarboxamide; OX1h, N-(3-methoxyphenyl)-2-thiophenecarboxamide; OX1i, N-(3-acetylphenyl)-2-thiophenecarboxamide; OX1j (136340-90-4), N-(2-ethylphenyl)-2-thiophenecarboxamide; OX1k (349097-45-6), N-(2,3-dimethylphenyl)-2-thiophenecarboxamide; OX1l, N-mesityl-2-thiophenecarboxamide; OX1m (853328-78-6), 3-chloro-N-(3-methylphenyl)-2-thiophenecarboxamide; OX1n, N-ethyl-N-phenyl-2-thiophenecarboxamide; OX1o (853328-84-4, 3-chloro-N-(2,4-dimethylphenyl)-2-thiophenecarboxamide; OX1p (853328-80-0), 3-chloro-N-(2,6-dimethylphenyl)-2-thiophenecarboxamide; OX1q (853328-88-8), 3-chloro-N-(3-chlorophenyl)-2-thiophenecarboxamide; OX1r, N-(4-propionylphenyl)-2-thiophenecarboxamide; OX1s, N-(4-butyrylphenyl)-2-thiophenecarboxamide; OX2, N-cyclohexyl-2-thiophenecarboxamide; OX3a, N-(4-methylbenzyl)-2-thiophenecarboxamide; OX3b, N-benzyl-2-thiophenecarboxamide; OX4 (853347-88-3), N-(2-ethylphenyl)-3-(2-thienyl)-2-propenamide. 

### Expression of Insect ORs in *Xenopus* Oocytes

Oocytes were surgically removed from mature *Xenopus laevis* frogs. Follicle cells were removed by treatment with Collagenase B (Boehringer Mannhem) for 2 hours at room temperature. Capped cRNA encoding each OR subunit was generated using mMessage mMachine kits (Ambion). For heteromeric ORs, 25 ng of cRNA encoding each OR subunit was injected into Stage V-VI *Xenopus* oocytes. For expression of Orco alone, 50 ng of cRNA was injected. Oocytes were incubated at 18°C in Barth's saline (in mM: 88 NaCl, 1 KCl, 2.4 NaHCO_3_, 0.3 CaNO_3_, 0.41 CaCl_2_, 0.82 MgSO_4_, 15 HEPES, pH 7.6, and 150 µg/ml ceftazidime) for 2–5 days prior to electrophysiological recording. 

### Electrophysiology and Data Capture

Odorant and Orco ligand induced currents were recorded under two-electrode voltage clamp, using an automated parallel electrophysiology system (OpusExpress 6000A; Molecular Devices). Oocytes were perfused with ND96 (in mM: 96 NaCl, 2 KCl, 1 CaCl_2_, 1 MgCl_2_, 5 HEPES, pH 7.5). Orco ligands were prepared as 50 or 100 mM stock solutions in DMSO and then diluted into ND96 on the day of the experiment. Unless otherwise noted, applications were for 60 sec at a flow rate of 1.0 ml/min, with extensive washing in ND96 at 4.6 ml/min between applications. For the concentration-response protocol in Figure 1d , applications were for 20 sec at a flow rate of 1.65 ml/min. Micropipettes were filled with 3 M KCl and had resistances of 0.2–2.0 MΩ. The holding potential was -70 mV. Current responses, filtered (4-pole, Bessel, low pass) at 20 Hz (-3 db) and sampled at 100 Hz, were captured and stored using OpusXpress 1.1 software (Molecular Devices).

### Experimental Protocols and Data Analysis

To screen for antagonist activity, oocytes were exposed to 60 sec applications of OLC12 with 4 min washes between applications (Figures 1B, 3, 4 and 5A,C,E) or 60 sec applications of odorant with 20 min washes between applications (Figures 1C,D and 5B,D,F). Oocytes were then exposed to a 90 sec application of antagonist candidate, immediately followed by a 60 sec co-application of antagonist candidate and OLC12 or odorant. Oocytes were then exposed to a final 60 sec application of OLC12 or odorant. An example trace illustrating this protocol is shown in Figure 3A. The current response in the presence of antagonist candidate was compared to the preceding responses to OLC12 or odorant alone.

Initial analysis of electrophysiological data was done using Clampfit 9.1 software (Molecular Devices). Curve fitting and statistical analyses were done using Prism 5 (Graphpad). Concentration-response data were fit to the equation: I = I_max_/(1+(EC_50_/X)^n^) where I represents the current response at a given concentration of odorant, X; I_max_ is the maximal response; EC_50_ is the concentration of agonist yielding a half maximal response; n is the apparent Hill coefficient. Concentration-inhibition data were fit to the equation: I = I_max_ / (1+ (X/IC_50_)^n^) where I represents the current response at a given concentration of inhibitor, X; I_max_ is the maximal response in the absence of inhibitor; IC_50_ is the concentration of inhibitor present that still allows a half maximal response from odorant; n is the apparent Hill coefficient. Statistical significance was assessed using a two-tailed unpaired *t* test, an F test, or a one-way analysis of variance followed by the Dunnett's post-test, as appropriate.

Boiling point and vapor pressure of selected compounds were estimated using the ACD/PhysChem Suite function avaliable on the Chemspider website (www.chemspider.com).
